# Medical Consequences of Alcohol Abuse

**Published:** 2000

**Keywords:** chronic AODE (effects of AOD [alcohol or other drug] use, abuse, or dependence), alcoholic liver disorder, immune system, cardiovascular system, bone, breast, cancer, alcoholic cardiomyopathy, heart disorder, cardiac arrhythmia

## Abstract

Studies have shown that long-term alcohol abuse produces serious, harmful effects on a variety of the body’s organ systems. Parts of the human body most affected include the liver and the immune, cardiovascular, and skeletal systems. Current research has examined some of these effects in an effort to better understand the medical consequences of alcohol use and abuse and to ultimately develop more effective treatments for responding to alcohol-induced bodily damage. This article discusses some of those findings.

Long-term alcohol abuse is known to exert harmful effects on a number of the body’s organ systems. Those most affected by alcohol abuse include the liver and the immune, cardiovascular, and skeletal systems. Many of the mechanisms involved in alcohol’s effects on these systems are not yet completely understood. Consequently, recent research has examined some of alcohol’s medical consequences in an effort to increase understanding and develop appropriate and effective treatments.

## Alcoholic Liver Disease

The liver is a vital organ involved in processing fats, sugars, proteins, and vitamins and in regulating blood clotting. It plays a central role in the body’s defenses, filtering toxins and microbes from the blood and marshaling an array of responses to trauma, stress, or inflammation.

Although the liver is capable of regeneration and repair, severe liver disease can be life threatening. Long-term, heavy alcohol use is the leading cause of illness and death from liver disease in the United States. The number of persons with alcoholic liver disease (ALD), which ranges in severity from fatty liver to end-stage cirrhosis, is conservatively estimated at more than 2 million. Women, compared with men, develop alcoholic hepatitis and alcoholic cirrhosis after fewer years of drinking and from ingesting smaller daily amounts of alcohol. Rat studies confirm greater liver damage in females than in males at the same blood alcohol concentration (BAC).

There are three forms of ALD: fatty liver, which is usually reversible with abstinence; alcoholic hepatitis, characterized by persistent liver inflammation; and cirrhosis, characterized by progressive scarring of liver tissue. A person can have more than one type of liver disease. Patients with both cirrhosis and alcoholic hepatitis have a death rate of more than 60 percent over a 4-year period, with most deaths occurring within the first 12 months of diagnosis.

The major problem in developing new therapies for ALD has been a lack of understanding of the mechanisms for liver injury. However, much has been learned recently as a result of better technology and advances in research.

### The Process of Inflammation

Long-term alcohol consumption has been shown to prolong the natural inflammatory responses of the liver. Inflammation functions to prevent the spread of localized injury or infection while mobilizing the defense mechanisms of the immune system. An important aspect of liver inflammation is the production of chemical messengers called cytokines, which help regulate the inflammatory process. Cytokines attract and activate cells of the immune system, promote scar formation, and stimulate the production of additional chemical messengers, including more cytokines. However, if the increased levels of cytokines do not subsequently return to normal, they can cause chronic inflammation, leading to cell injury or cell death.

Cytokine production can be stimulated by endotoxin, a substance derived from the cell walls of certain bacteria that reside in the human intestine. Heavy alcohol consumption can increase the passage of endotoxin through the intestinal wall into the bloodstream. Specialized immune system cells (i.e., Kupffer cells) in the liver respond to blood-borne endotoxin by producing inflammatory cytokines. These cytokines further increase gut permeability, perpetuating a destructive cycle.

Another stimulus for excessive cytokine production is the generation of reactive oxygen species (ROS), toxic by-products of alcohol metabolism in the liver. Normally, ROS are quickly inactivated by antioxidants, protective molecules such as glutathione and vitamins A and E. However, if these defenses are impaired or if there is an overproduction of ROS, the result can be destruction of cell components and eventual cell death.

Persistent liver inflammation is characteristic of alcoholic hepatitis and usually precedes alcoholic cirrhosis. The hallmark of cirrhosis is cytokine-induced scarring that distorts the liver’s internal structure and impairs its function. Imbalances in cytokine interactions impair the normal regeneration of tissue that typically follows liver injury.

### Preventing Alcoholic Liver Injury

Researchers have devised several strategies to prevent or minimize alcoholic liver damage. In experiments using rats, alcohol-induced liver injury has been lessened by suppressing endotoxin-producing intestinal bacteria (e.g., through the use of antibiotics); administering substances that selectively destroy Kupffer cells or that inhibit the generation of ROS; and feeding antibodies that neutralize specific cytokines.

Experiments with a soybean extract, polyenylphosphatidylcholine (PPC), showed that it could prevent fibrosis and cirrhosis in alcohol-fed baboons. It also reduces the formation of scar tissue and may possess antioxidant properties as well. A study conducted by the U.S. Department of Veterans Affairs is currently evaluating the effects of PPC in humans with early ALD. Therapy with S-adenosyl-l-methionine (SAM) may lessen the depletion of the antioxidant glutathione in liver cells. Choline and methionine, dietary factors related to SAM, may help protect against endotoxin-induced liver injury in rats.

## Alcohol’s Effects on the Immune System

For 200 years physicians have observed that excessive alcohol consumption can lead to increased illness and death from infectious diseases. Alcohol abusers suffer from increased susceptibility to bacterial pneumonia, pulmonary tuberculosis, and hepatitis C (HCV). Patients with ALD are at high risk of having HCV, the leading cause of liver transplantation in the United States (National Institute on Alcohol Abuse and Alcoholism [NIAAA] 1998). Findings indicate that patients with ALD who are HCV positive have more severe liver disease and are younger than HCV-negative patients (NIAAA 1998). This increase in disease may reflect impaired immune function (i.e., immunodeficiency) caused by alcohol abuse.

Alcohol abusers may be at increased risk compared with nonabusers for infection with human immunodeficiency virus (HIV) from risky sex practices while intoxicated. Researchers also are investigating whether alcohol consumption itself may increase susceptibility to HIV infection or hasten the progression from HIV infection to full-blown AIDS.

In addition, some alcohol-related organ damage, as in ALD, may result in part from immune system overactivity in which the immune system attacks the body’s own tissues (i.e., autoimmunity). Current research is examining the effects of heavy drinking on the immune system.

### How the Immune System Works

The body’s first line of defense against disease is inflammation, as described earlier. Inflammation is a nonspecific response, directed against all sources of damage. When nonspecific defenses are breached, an array of specific immune responses (see table) come into play. Specific immune responses may be broadly classified as either cell-mediated or humoral. Cell-mediated immunity involves direct contact between immune system cells and target cells (e.g., bacteria). Humoral immunity is provided by antibodies that circulate in the blood and lymph. Antibodies are specialized proteins designed to recognize and disable specific microoganisms or toxic substances. Antibodies that persist in the bloodstream may confer long-term immunity to a given disease.

Phagocytes are specialized cells that engulf invading microorganisms and cell debris through a process called phagocytosis. The first phagocytes to be activated are macrophages, which reside in tissues and organs throughout the body. White blood cells infiltrate the area, with neutrophils (a type of phagocyte) being the first to arrive, followed by monocytes. Monocytes are one of the types of cells that initially determine the “foreignness” of an invading agent and present it to other cells, which respond by producing appropriate cytokines. Various immune cells release chemical messengers to attract more cells, and within neutrophils and macrophages, the toxic oxygen-containing compounds ROS destroy phagocytosed microorganisms. Excess ROS, however, can damage liver cells.

Throughout the immune response, cytokines continue their regulatory functions. They include tumor necrosis factor (TNF) and a family of both inflammatory and anti-inflammatory substances called interleukins.

### How Alcohol Affects the Immune System

Both chronic and acute alcohol administration can produce the loss of various types of immune responses in experimental animals. Administration of alcohol concentrations similar to those seen in binge drinkers impairs the function of cultured human monocytes and can temporarily reduce the numbers and activity of immune cells in mice. Alcohol inhibits neutrophil migration in humans and in experimental animals. However, an infiltration of neutrophils into the liver is observed in alcoholic hepatitis. Experiments with alcohol-fed rats showed that their neutrophils engulfed bacteria efficiently but did not kill all strains of pneumonia-causing bacteria with normal effectiveness.

Although inflammatory cytokine levels increase in ALD, endotoxin-induced secretion of inflammatory cytokines in the lung may decrease in alcohol-fed animals as well as in human alcoholics, potentially increasing susceptibility to pneumonia. Acute administration of alcohol to rats reduced ROS production by isolated lung macrophages after challenge with TB organisms.

### Therapeutic Measures

Some proposed therapies include administration of such substances as antibodies against endotoxin or against specific cytokines, substances that would absorb excess cytokines or inhibit their function, and drugs that have a widespread effect (e.g., decreasing the permeability of the intestinal wall to the passage of endotoxin). Administration of growth hormone and related chemical messengers has been shown to improve some, but not all, measures of immune function in rats.

## Alcohol’s Effects on the Cardiovascular System

Chronic heavy drinking is a leading cause of cardiovascular illnesses such as degenerative disease of heart muscle (cardiomyopathy); disorders associated with decreased blood supply to the heart muscle (coronary heart disease [CHD]); high blood pressure; heart rhythm disorders (arrhythmias); and stroke.

### Alcoholic Cardiomyopathy

Long-term heavy drinking can cause the heart to become enlarged and lose some of its ability to contract, a condition known as alcoholic cardiomyopathy. These symptoms include shortness of breath and an insufficient blood flow to the rest of the body. Women may have a greater risk than men of developing alcoholic cardiomyopathy. The condition may be at least partially reversible with abstinence.

Alcohol’s toxic effects on heart muscle may be mediated by increased ROS levels and decreased antioxidant enzyme activity. Another recent study found that alcohol may decrease the sensitivity of heart muscle to chemical messengers from nerve cells that regulate heart muscle metabolism and contraction.

### Coronary Heart Disease

The blood that nourishes the heart muscle is delivered through the coronary arteries. Manifestations of CHD range from episodic chest pain to sudden death. Heart attacks, the most common serious manifestation of CHD, are usually triggered by the formation of a blood clot within a coronary artery already narrowed by deposits of cholesterol and other fatty substances. The resulting ischemia reduces the heart’s pumping ability, often leading to permanent disability or death.

With few exceptions, worldwide epidemiologic data demonstrate a 20- to 40-percent lower CHD incidence among drinkers compared with non-drinkers. Heavy drinkers have an increased risk of death from heart disease. However, moderate drinkers exhibit lower rates of CHD-related mortality than both heavy drinkers and abstainers. This is confirmed by studies in which participants were interviewed about their drinking habits and life styles before the onset of disease. Such studies—representing a total population of more than 1 million men and women of different ethnicities followed for up to 24 years—confirm an association between moderate drinking and lower CHD risk. However, this association does not necessarily mean that alcohol itself is the cause of the lower risk.

In addition, different epidemiologic studies apply the term “moderate drinking” to a wide range of consumption levels, sometimes more than the amount defined by the Dietary Guidelines for Americans as moderate: two or fewer standard drinks per day for men and one or less per day for women. In any case, the apparent benefits of moderate drinking on CHD mortality are offset at higher drinking levels through increasing risk of death from other alcohol-related causes.

Research has suggested several possible mechanisms by which alcohol may protect against CHD. For example, alcohol inhibits the deposition of fatty substances within the coronary arteries of mice and also may inhibit the formation of blood clots within already narrowed coronary arteries.

Epidemiologic data and results of studies on isolated animal hearts suggest that moderate alcohol consumption also may lower CHD mortality by improving survival after a heart attack. Further studies are needed to confirm this effect and to determine its applicability in humans.

### Alcohol and Blood Pressure

An association between heavy alcohol consumption and increased blood pressure has been observed in more than 60 studies in diverse cultures and populations. The effects of moderate alcohol consumption on blood pressure are unclear.

### Arrhythmias

The heart’s ability to function effectively depends on regular, synchronous contraction of the heart muscle. Heavy drinking can disrupt the heart rhythm both acutely (during an episode of drinking) and chronically (due to long-term use). Intoxication can cause certain types of arrhythmia in both alcoholics and otherwise healthy persons. The development of arrhythmias with binge drinking—a condition seen most frequently around the holidays—is known as “holiday heart syndrome.”

Sudden death attributable to arrhythmia is one of the causes of mortality in alcoholics with or without pre-existing heart disease. Such deaths often occur during periods of abstinence (Clark 1988), suggesting the development of arrhythmias during alcohol withdrawal.

### Alcohol and Stroke

The relationship between alcohol consumption and stroke is similar to that seen with CHD. Moderate alcohol consumption appears to be associated with lower incidence of ischemic strokes, whereas heavy drinking may increase the risk of both ischemic and hemorrhagic strokes (i.e., bleeding within the brain).

## Alcohol and Bone

Epidemiologic studies have found a significant association between alcohol consumption and risk for bone fracture. In addition to increased risk of accidental injury through alcohol-induced impairment of gait and balance, alcoholics also may suffer from a generalized decrease in bone mass, making their bones more fragile. Heavy drinking may lead to osteoporosis, characterized by severe back pain, spinal deformity, and increased risk of wrist and hip fractures, although some recent studies suggest that moderate alcohol consumption may protect against osteoporosis.

### Alcohol and Skeletal Development

Bone growth and remodeling during childhood and adolescence involves the coordinated activity of two types of cells: (1) osteoclasts, which break down (i.e., resorb) bone, and (2) osteoblasts, which form new bone.

Cyclic bone remodeling continues throughout adulthood to regulate and maintain bone mass. Adult bone formation and resorption rates are tightly coupled, with approximately 10 percent of bone undergoing the process at any given time. Between ages 20 and 40, a person’s bone density begins to decline, resulting in a cumulative decrease in skeletal mass of up to 40 percent by age 70. Women experience accelerated decline in bone density following menopause and are more susceptible than men to osteoporosis.

Alcohol disrupts bone remodeling in animals and humans. Overall, studies in alcoholics suggest that alcoholic bone disease involves considerable suppression of bone formation with essentially normal rates of resorption. The changes in bone turnover induced by alcohol can apparently be reversed by abstinence. The skeletal consequences of alcohol intake may be especially harmful during adolescence, when the rapid skeletal growth ultimately responsible for achieving peak bone mass occurs. By limiting peak bone mass attainment, the risk of developing osteoporosis later in life may increase and its onset hastened.

### Potential Mechanisms of Alcohol-Induced Bone Disease

Long-term consumption of alcohol disrupts the processes of bone growth and bone tissue repair. A decrease in bone density, as well as an increased risk of bone fracture, may result. These effects of alcohol on bone may occur directly, with alcohol itself interfering with bone metabolism, or indirectly, with alcohol exerting its effects through a third party, such as hormones. The female reproductive hormone estrogen appears to affect bone metabolism, although its role with respect to alcohol consumption is uncertain.

A number of researchers have noted that alcohol can reduce osteoblast formation. Alcohol can inhibit osteoblast proliferation in culture at concentrations well within the drinking level observed in alcoholics. A concentration of alcohol equivalent to a blood alcohol level of 0.044 percent, about half the blood alcohol level that many States define as legally intoxicated, also resulted in a 20-percent decline. Alcohol also may depress osteoblast function by inhibiting the cell’s response to insulin-like growth factors, chemical messengers that help regulate bone remodeling.

Studies of alcohol’s effects on osteoclast numbers have provided conflicting results. However, alcohol increases levels of a specific interleukin (IL-6), which may contribute to the development of osteoporosis by stimulating osteoclastic activity.

## Breast Cancer

The lifetime risk for breast cancer among U.S. women is estimated to be as high as one in eight. Results of approximately 50 epidemiologic studies and analyses conducted since the 1970s point to an increase in breast cancer risk associated with alcohol consumption. Controversy remains over the interpretation of these studies, however. For epidemiologists, the actual numerical association between alcohol and breast cancer risk is considered relatively modest. In addition, some studies found no link between high alcohol intake and breast cancer risk.

### Mechanisms of Alcohol-Related Breast Cancer

Scientists have identified plausible biological mechanisms for alcohol’s actions. Research findings suggest a role for alcohol in breast cancer risk in both premenopausal and postmenopausal women. Cumulative lifetime exposure to estrogen is considered an important contributor to breast cancer risk. A number of studies have examined whether alcohol raises estrogen levels in premenopausal and postmenopausal women. Although some studies report such an effect, the evidence is not conclusive.

Alcohol may be capable of enhancing the progression as well as the initiation of cancer. Increased metastasis (proliferation beyond the site of origin) of implanted breast cancer cells was observed in rats given alcohol in a liquid diet. In addition, alcohol and its highly reactive metabolite acetaldehyde also have been linked to the body’s inability to repair damage to the cell’s genetic material (i.e., DNA) induced by cancer-causing agents. If unrepaired, damage to critical regions of DNA in breast cells could lead to mutations and the subsequent initiation of cancer. When rodents are fed alcohol, their levels of circulating prolactin—a hormone that can stimulate the growth of breast tissue—increase. In addition, ROS can contribute to tumor promotion. Alcohol intake also decreases the immune system’s ability to detect and destroy cancer cells (Yirmiya and Taylor 1993).

## Figures and Tables

**Table:** Mediators of the Immune Response

Immune Response	Function
**Humoral**	
Antibody	A specialized protein that recognizes and disables specific microorganisms or toxic substances. When persisting in the bloodstream, it may help confer long-term immunity against a specific disease.
**Cell-Mediated**	
Phagocyte	A specialized cell that engulfs invading microorganisms and cell debris through a process known as phagocytosis.
Macrophage	A type of phagocyte that is the first to be activated during phagocytosis. It resides in tissues and organs throughout the body.
Neutrophil	A type of phagocyte that aids in the destruction of invading microorganisms, in part by releasing toxic compounds known as reactive oxygen species (ROS). Together with monocytes, neutrophils are commonly referred to as white blood cells.
Monocyte	A type of cell that is responsible for determining the “foreignness” of an invading agent and then presenting such agents to other cells for destruction. Monocytes release ROS to aid in the destruction process. They are also referred to as white blood cells.
